# Yield-based drought tolerance index evaluates the drought tolerance of cotton germplasm lines in the interaction of genotype-by-environment

**DOI:** 10.7717/peerj.14367

**Published:** 2023-01-10

**Authors:** Fenglei Sun, Quanjia Chen, Qin Chen, Menghui Jiang, Yanying Qu

**Affiliations:** College of Agronomy, Xinjiang Agricultural University, Urumqi, China

**Keywords:** Cotton, Yield index, Drought resistance, Cotton yield per plant, Water stress

## Abstract

**Background:**

Cotton is an economically important crop in China, and drought has seriously affected cotton production. Understanding genetic variation, genotype ×environment interactions, and the associations between these traits is critical for developing improved cotton varieties with high drought tolerance.

**Methods:**

To screen ideal drought-resistant cotton germplasm lines and excellent genotypes, the yield traits of 103 cotton germplasm lines were analyzed. Cotton resource material was planted under normal watering and water deficit conditions for three consecutive years. The yield traits under normal irrigation and water stress conditions were measured, and then five screening indicators were calculated based on the cotton yield per plant under the two water treatments to determine the ideal genotype and most accurate identification indicators.

**Results:**

The results of correlation analysis and principal component analysis showed that the geometric mean productivity (GMP), mean productivity (MP), and stress tolerance index (STI) were significantly positively correlated with yield under water stress and could be used to distinguish genotypes with high drought tolerance. Among the experimental germplasm lines, some had higher STI and GMP values, indicating their higher drought tolerance. This result indicates that best linear unbiased prediction (BLUP) analysis of the STI and GMP under drought stress can effectively improve screening for drought tolerance in cotton germplasm lines. The results from the screening index, three-dimensional map, and genotype ×environment (GGE) biplots were consistent with the above results. We determined that CQJ-5, Xin lu zao 45, Bellsno, Zhong R 2016 and ND 359-5 are drought-tolerant genotypes that can be used to breed drought-tolerant germplasm lines that produce high and stable yields.

## Introduction

Water stress is an abiotic stress that is primarily responsible for low crop yields in many countries and regions around the world (Abdelraheem et al., 2019; Sinclair, 2005). Cotton is a drought-sensitive crop that is most sensitive to water stress during flowering and fruit development (Wang et al., 2010; Abdelraheem, Fang & Zhang, 2017; Turner et al., 1986). Northwest China faces severe water shortages, severe droughts, and increasing desertification (Ling et al., 2013). One key sustainability issue in this region is the shortage of water for agricultural production (Zhou et al., 2012). Xinjiang in Northwest China is an arid and semiarid area with high evaporation and a general shortage of freshwater resources. Cotton is the main economic crop in Xinjiang in the arid regions of Northwest China, accounting for more than one-third of the total agricultural area in the region (Wang, Isoda & Wang, 2004; Kang et al., 2012). At present, the cotton planting area in Xinjiang accounts for 70% of the cotton planting area in China, and the total output accounts for 84% of the national total (source: National Bureau of Statistics of China (2020)). Drought restricts plant growth by causing changes in metabolic activity and biological functions, which are ultimately manifested through yield (Mahmood et al., 2020). Cotton yields 34% lower due to drought (Ullah et al., 2017). Therefore, cotton varieties that can produce high yields under drought conditions are currently the main breeding target in this region (Cattivelli et al., 2008).

To breed crop materials, two requirements need to be met. First, there must be a difference in water stress tolerance at the overall level of the crop; second, this variation must be genetically controlled (Volkan et al., 2015; Areej et al., 2015). Breeding drought-tolerant cotton varieties requires a large number of materials to be screened. Drought resistance is genetically related to various morphological and physiological characteristics of crops (Singh, 2004). Among abiotic stresses, drought has the greatest impact on cotton growth and development, and it severely limits cotton yield and fiber quality (Pettigrew, 2004; Wiggins et al., 2013). The flowering and boll development stages are the key irrigation stages that determine the yield of upland cotton (*Gossypium hirsutum L.*) (Han & Kang, 2001). At present, 30 traits have been proposed as important indicators that are related to drought tolerance in cotton (Loka, Oosterhuis & Ritchie, 2011), but these indicators have not been positively correlated with drought tolerance consistency (Loka, Oosterhuis & Ritchie, 2011). Water stress will affect crop morphological characteristics and yield (Soomro, Markhand & Soomro, 2011). Therefore, the use of major component traits as selection criteria has been recommended (Munir, Chowdhry & Ahsan, 2007).

In the last two decades, many methods have been used to evaluate the drought tolerance of crops. Yield has been the most commonly used criterion for evaluating cotton drought tolerance. Some researchers have used mathematical models to compare changes in cotton yield under water stress and normal irrigation conditions (Reynolds, Mujeeb-Kazi & Sawkins, 2005; Yadav & Bhatnagar, 2001). Fernandez (1992) defined a stress tolerance index (STI) that can be used to identify genotypes that produce high yields under stressed and nonstressed conditions. The irrigation environment can also be considered. The mean productivity (MP) is calculated as the average yield of genotypes under stressed and nonstressed conditions (Rosielle & Hamblin, 1981). Geometric mean productivity (GMP) is also often used as an indicator of drought tolerance (Kristin et al., 1997).

Genotype and genotype × environment (GGE) biplots are widely used in multienvironmental data analysis (Yan et al., 2001). GGE biplots are based on phenotypic data to analyze the effects of environment (G), genotype (E), and G ×E interactions (Yan et al., 2000). GGE biplots developed with principal component analysis (PCA) can graphically display the performance of a genotype through its phenotypic value and can accurately reflect the yield and yield stability of the test material (Yan et al., 2007).

In this study, yield performance under drought conditions was used as the main indicator of drought tolerance (Voltas, Lopez-Corcoles & Borras, 2005), and some significant features were selected as effective and efficient indicators to identify drought tolerance in large populations and improve screening efficiency (Cetin et al., 2009). Therefore, this study evaluates the effectiveness and accuracy of the GMP, MP, STI, stress susceptibility index (SSI), and the maximum yield under normal conditions (Yr) in combination with yield trait for assessing drought tolerance in different cotton varieties. The screening index analysis in this study provides a theoretical basis for cotton drought tolerance breeding practices.

## Materials and Methods

A total of 103 cotton germplasm lines that were collected and preserved by the Crop Genetics and Breeding Laboratory of Xinjiang Agricultural University were used for drought tolerance identification. This study was carried out in the cotton breeding laboratory test field (43°20′∼45°20′E, 84°45′∼86°40′N) of the Xinjiang Agricultural University Experiment Station for three consecutive years (2016–2018). The average elevation of this area is 300-500 m, and it belongs to a temperate continental climate. The annual average temperature is 7.5−8.2 °C, the sunshine duration is 2318–2732 h, and the frost-free period is 147-191 days. The temperature and precipitation data statistics are shown in Table S1. The soil contained 0.23 g/kg available phosphorus, 0.29 g/kg available potassium and 0.33 g/kg total nitrogen, with a pH of 8.3. The details of the 103 cotton germplasm lines are shown in Table S2.

All germplasm lines were planted in the same experimental area, and the experimental area was divided into two parts before planting: one had normal watering (CK), and the other had drought stress (DS). Each material was planted in two rows, where the row length was 3 m, the distance between the two germplasm lines was 50 cm, and the spacing between the plants in each plot was 10 cm. Drought stress treatment was achieved by manually controlling water during the growth of cotton plants (irrigation was stopped in the stress treatment). The time of drought stress treatment was during the flowering and boll-setting period of cotton, the control group was watered normally, and the stress group was not watered twice. After undergoing stress treatment, the soil water content of the drought stress-treated plots was measured, and the water content was approximately 45–60% (Table S3), which met the stress treatment conditions. All experiments were designed with a completely randomized block design, with twice replicates per treatment, separated by guard rows.

After maturity in early October, yield traits were investigated, including the yield of each material in each treatment. Twenty bolls were collected from the middle of the cotton plant in the selected area to determine the total weight, and the yield per plant was calculated. Then, the yield per plant of each material under the two treatments was estimated. The main investigation methods were performed based on the “Cotton Germplasm Description Specifications and Data Standards” (Du & Zhou, 2005).

### Data analysis

High-yield classification is the best indicator for assessing drought tolerance (Ramirez & Kelly, 1998). Therefore, the yield and drought tolerance of the tested germplasm lines were evaluated by determining their changes in yield under water stress. The yields of crop species under control were reduced by environmental factors other than water stress (Golestani-Araghi & Assad, 1998; Zhao et al., 2019). Four drought tolerance indices, GMP, MP, STI, SSI and Yr were calculated as:

\begin{eqnarray*}\mathrm{GMP}& =\sqrt{ \left( Ys\ast Yp \right) } \end{eqnarray*}

\begin{eqnarray*}\mathrm{MP}& = \frac{(Yp+Ys)}{2} \end{eqnarray*}

\begin{eqnarray*}\mathrm{STI}& = \frac{Yp\ast Ys}{\overline{ \left( Yp \right) 2}} \end{eqnarray*}

\begin{eqnarray*}\mathrm{SSI}& = \frac{1- \left( Yp+Ys \right) }{1-\bar {Yp}+\bar {Ys}} \end{eqnarray*}

\begin{eqnarray*}\mathrm{Y r}& =1-(\mathrm{Y s}/\mathrm{Y p}) \end{eqnarray*}

where Ys is the yield under water stress and Yp is the yield under normal irrigation conditions.

An analysis of variance was performed on cotton yield and the drought tolerance indices using SSPS 21.0. Correlation analysis was used to determine the correlation between the seven screening indicators and cotton yield under stress conditions. Three-dimensional (3D) maps were draw with R software to identify the genotypes with strong drought resistance and high yield. The PCA and GGE biplots based on the indices and yield of the three environments were drawn using R. The best linear unbiased prediction (BLUP) value of the cotton yield genotype effect under different drought stress treatments was evaluated with R (lme4 package), and the mixed linear model of each cultivar was as follows (He et al., 2018):

\begin{eqnarray*}yi=\mu +Gi+Ei+ei \end{eqnarray*}

where yi = phenotypic value; µ= total mean value of the total yield in all environments; Gi = genotype effect; Ei = environment effect; and ei is the random error. The genotype effect and environmental effect were random effects, and the others were assumed to be fixed (He et al., 2018).

The GGE biplots model is shown below (Yan et al., 2000):

\begin{eqnarray*}Yij-\mu -\beta j={\lambda }_{1}{\xi }_{\mathrm{i1}}{\ng }_{\mathrm{j1}}+{\lambda }_{2}{\xi }_{\mathrm{i2}}{\ng }_{\mathrm{j2}}+ij \end{eqnarray*}

where Yij is the expected yield (i is the genotype, j is the environment); µis the grand mean; *β*j is the environmental main effect; *μ* + *β*j is the mean yield in the environment; *λ*_1_ and *λ*_2_ are the singular values (SV) of PC1 and PC2, respectively; *ξ*_i1_ and *ξ*_i2_ represent the eigenvectors of PC1 and PC2, respectively; ŋ_j1_ and ŋ_j2_ are the eigenvectors of PC1 and PC2 in different environments; *ɛ*ij refers to residuals (i refers to genotype, j refers to environment) (Yan et al., 2000).

## Results

### Phenotype, yield per plant and values of the eight screening indicators for 103 natural cotton populations under normal and stress treatments

The results for the different screening indicators calculated as the BLUP value for cotton plant yield are shown in Table 1. Compared with that under normal conditions, the yield per plant of all cotton germplasm lines under drought stress decreased to varying degrees (Table 1, Table S6). GMP, MP, STI, and SSI confirmed that the genotypes Bellsno, Shi yuan 321, ND359-5 and Xin lu zao 36 were the most tolerant. The genotypes Shuo feng 1 hao, Ji zha 81, and Hong ye mian were identified as having the least sensitivity in terms of GMP and STI (Table 1, Table S6). The results of the analysis of variance of the combined data for the yield per plant and screening indices for 103 cotton germplasm lines are presented in Table 2. We found significant differences among the three growing years and different genotypes. There was a significant difference in yield per plant and all screening indicators among all genotypes (*p* < 0.01), which indicated that the different germplasm lines performed differently regarding yield per plant and all screening indicators. The differences among all genotypes under the stress treatment provided the basic screening indicators.

**Table 1:** The best linear unbiased prediction (BLUP) value of the best yield under normal (Yp) and stress treatments (Ys) and the values of the eight screening indices for some of the cotton materials.

Genotype	Yp (g/plant)	Ys (g/plant)	GMP	MP	STI	SSI	Yr
Bellsno	38.88	25.46	31.24	32.46	0.81	4.94	0.35
Chuan 98	39.21	20.34	28.06	30.84	0.65	4.69	0.37
CQJ-2	37.72	20.74	25.96	28.86	0.57	4.38	0.35
CQJ-5	39.49	26.77	33.22	33.86	0.89	5.16	0.35
ND359-5	39.33	24.34	31.02	32.64	0.74	4.97	0.35
Hong ye mian	36.17	20.79	23.19	26.65	0.52	4.04	0.33
KK1543	39.46	23.44	30.59	32.46	0.76	4.94	0.36
Ji zha 81	35.80	20.00	22.36	25.79	0.50	3.91	0.33
Shi K8	38.64	23.43	29.29	31.28	0.69	4.76	0.35
Shi yuan 321	39.55	23.93	31.10	32.79	0.79	4.99	0.35
Shuo feng 1 hao	37.29	18.77	22.68	27.43	0.48	4.16	0.33
Tian yun 10	39.11	24.47	31.05	32.39	0.80	4.93	0.35
Tai yuan 112	38.48	28.53	32.18	33.13	0.82	5.04	0.32
Tu 76-94	36.89	25.72	26.21	29.59	0.65	4.50	0.33
Xi bu 50	38.57	18.13	24.46	29.03	0.55	4.41	0.37
Xin lu zao 36	38.55	25.31	30.90	31.93	0.75	4.86	0.35
Xin lu zao 45	40.04	28.31	34.68	35.28	0.97	5.38	0.34
Xin nong mian 3	37.36	24.01	27.68	29.68	0.63	4.51	0.33
Zhong R 2016	39.58	25.56	32.68	33.53	0.88	5.11	0.35
Zhong R 2069	36.65	25.53	27.45	29.27	0.62	4.45	0.30
Zhong mian 49	37.39	18.27	23.48	27.33	0.50	4.15	0.36
Zhong mian suo 50	39.10	23.49	30.17	31.98	0.77	4.87	0.35
08207-2	37.36	24.55	28.15	29.82	0.66	4.53	0.33
10599	39.09	23.07	30.06	31.78	0.70	4.84	0.35
10615-1	37.90	22.85	28.03	29.98	0.68	4.56	0.35
108 Fu	37.93	22.67	27.72	29.94	0.63	4.55	0.34
2 hao	38.68	21.94	28.59	30.74	0.66	4.67	0.36
5917-N10-1	39.46	23.88	31.21	32.64	0.77	4.97	0.36
806-1	38.72	24.17	30.21	31.71	0.75	4.82	0.35
8401	38.99	20.15	26.99	30.44	0.60	4.63	0.36

**Notes.**

Yp yield under normal irrigation conditions Ys yield under water stress GMP geometric mean productivity MP mean productivity STI stress tolerance index SSI stress susceptibility index Yr yield reduction ratio

**Table 2:** Analysis of variance of cotton plant BLUP under normal (Yp) and stress treatments (Ys).

Source of variation	DF	Yp	Ys	STI	MP	GMP	SSI	Yr
Genotype (G)	102	254.61^**^	142.54^**^	0.30^**^	133.68^**^	146.87^**^	3.19^**^	0.15^**^
Year (Y)	2	22067.84^**^	16254.08^**^	24.10^**^	17205.24^**^	16678.56^**^	410.99^**^	4.83^**^
G*Y	203	230.68^*^	89.70^*^	0.19^*^	91.73^*^	84.14^*^	2.19^*^	0.14^*^

**Notes.**

Yp yield under normal irrigation conditions Ys yield under water stress GMP geometric mean productivity MP mean productivity STI stress tolerance index SSI stress susceptibility index Yr yield reduction ratio

* Significance at the *P* < 0.05 level.

** Significance at the *P* < 0.01 level.

### Correlation analysis between seven indices across germplasm lines and yield per plant after drought stress

**Figure 1:**
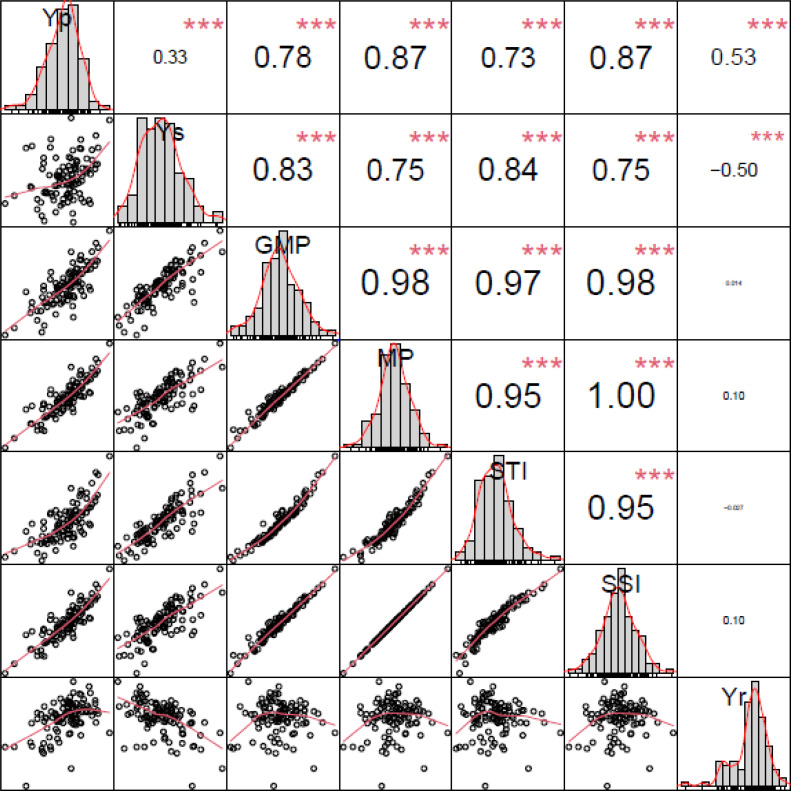
Correlation coefficients between the yield per plant (Yp and Ys) of 103 cotton materials and the values of five screening indicators. Yp, yield under normal irrigation conditions; Ys, yield under water stress; GMP, geometric mean productivity; MP, mean productivity; STI, stress tolerance index; SSI, stress susceptibility index; Yr, yield reduction ratio; asterisks (***) indicate significance at the *P* < 0.01 level.

The most effective or best indicators under drought conditions were identified as a method for evaluating ideal drought-resistant genotypes. The BLUP values of 103 germplasm lines under drought and normal conditions were calculated, and the correlation among the 7 yield indicators was analyzed. The correlation analysis results showed that Yp had a very significant positive correlation with GMP (*r* = 0.78), MP (*r* = 0.87), STI (*r* = 0.73), SSI (*r* = 0.87) and that Ys had a very significant positive correlation with GMP (*r* = 0.83), MP (*r* = 0.75), STI (*r* = 0.84), and SSI (*r* = 0.75). These results show that GMP, MP, STI, and SSI can be used to identify stable and high-productivity genotypes under normal and drought stress conditions. The several production indicators were divided into two main categories, among which GMP, MP, and STI were used to identify high-yielding lines, and SSI was used to identify stable-yielding lines. The GGE biplot was drawn in R, and the results showed that GMP, MP, STI and SSI were positively correlated with Yp and Ys (Fig. 1). This is consistent with the results of the correlation analysis.

### Principal component analysis of five evaluation indices and yield indices under drought stress

**Figure 2:**
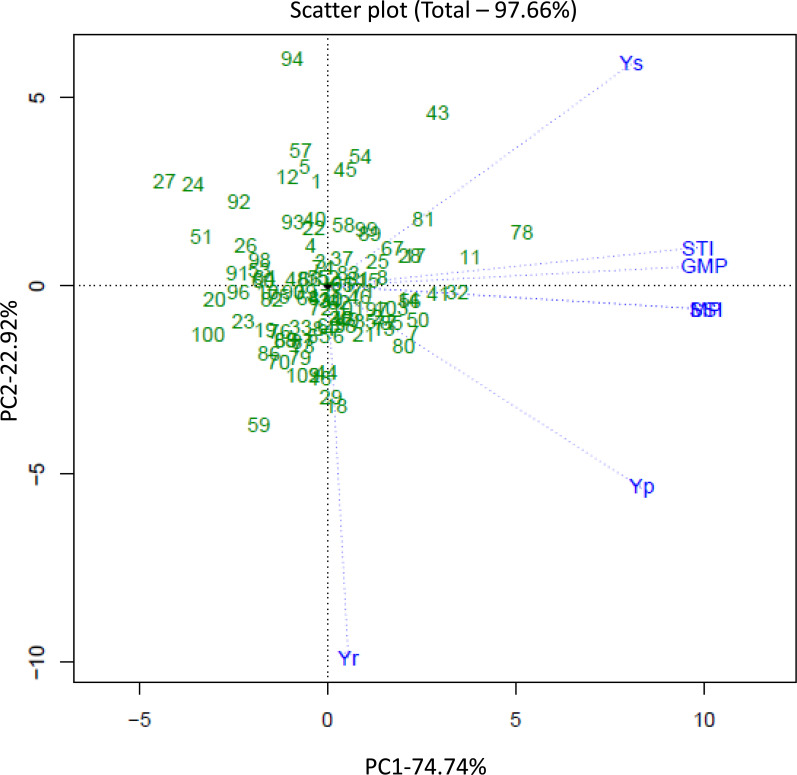
Genotype and genotype × environment (GGE) biplots based on the first (PC1) and second (PC2) principal components of 103 cotton materials. Yp, yield under normal irrigation conditions; Ys, yield under water stress; GMP, geometric mean productivity; MP, mean productivity; STI, stress tolerance index; SSI, stress susceptibility index; Yr, yield reduction ratio.

The principal components of each index were analyzed according to the cotton yield amounts and yield indices. The principal components were able to explain 97.66% of the total variation in cotton yield under the different moisture conditions (Table S5, Fig. 2). GGE biplots were drawn based on the BLUP values for Yp, Ys, GMP, MP, STI, and SSI, and the eigenvalues of the first two components were >1. The first principal component explained 74.74% of the variation in yield, and Yp, Ys, GMP, MP, STI, and SSI were closely related (Fig. 2). This indicates that these indices can be used to identify genotypes that will produce high yields under drought stress and normal conditions. The second principal component explained 22.92% of the variation and was correlated with Yr under normal conditions. Five germplasm lines CQJ-5, Xin lu zao 45, Bellsno, Zhong R 2016 and ND 359-5 had high values of PC1 (Fig. 2), which reflected their performance under drought conditions and indicated that they have high drought tolerance.

### Yield and 3D STI graphs of 103 germplasm lines under normal conditions and drought stress conditions

**Figure 3:**
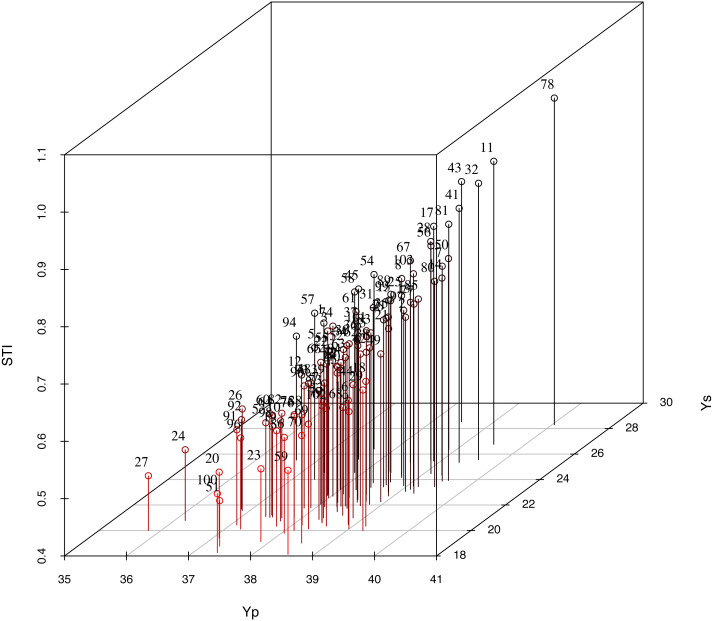
Three-dimensional map used to screen for drought-tolerant genotypes based on Ys, Yp and STI. Yp, yield under normal irrigation conditions; Ys, yield under water stress; STI, stress tolerance index.

Of the indices considered, STI had very significant positive correlations with Yp and Ys, and the cotton varieties exhibited stable STI values in production. Based on STI, Ys and Yp, 3D maps were drawn to identify the characteristics of 103 germplasm lines (Fig. 3). According to the 3D graph analysis, CQJ-5, Xin lu zao 45, Bellsno, Zhong R 2016 and ND 359-5 were determined to have stable yield in terms of the STI indicator. These genotypes had higher STI values than the other genotypes and were located in the first quadrant of the 3D graph, indicating that they produce higher yields under drought conditions and normal conditions. These results show that these genotypes have a more stable, higher yield capacity under stress and normal conditions. These results demonstrate the function of the screening index for evaluating cotton genotypes.

### GGE biplots for the seven indices and yield of 103 germplasm lines under drought and normal conditions

**Figure 4:**
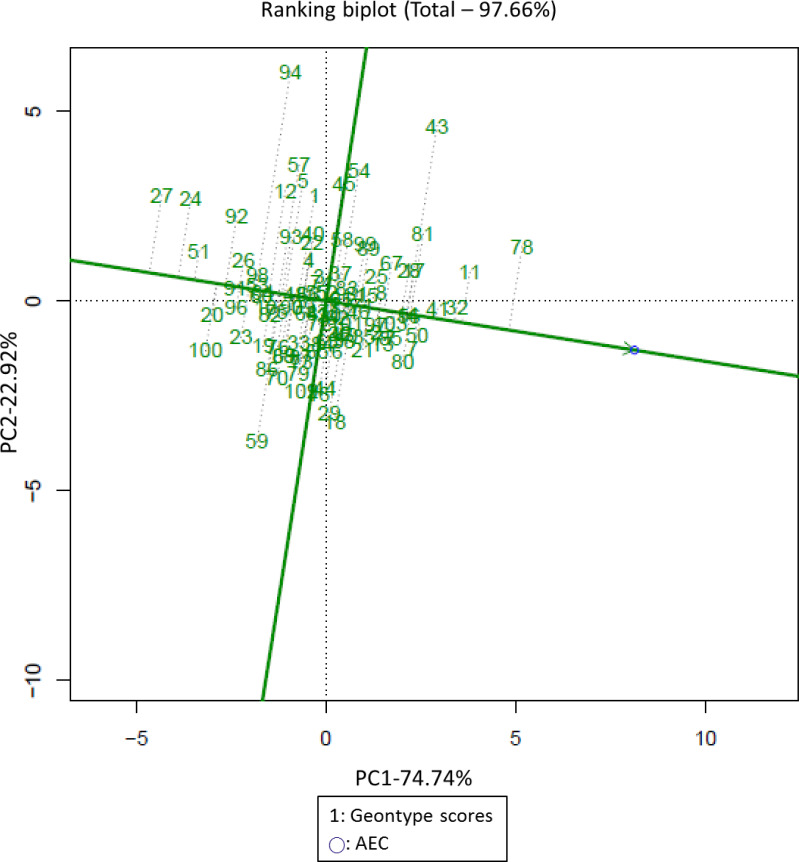
GGE biplots of stable-yield and high-yielding cotton material. Numbers represent germplasm line serial number, is AEC, PC1 refers to the first principal component, PC2 refers to the second principal component.

The yield stability of the 103 cotton germplasm lines is shown in the figure. The Yp, Ys and all indicators for the two conditions (stress and normal) were used to draw the GGE biplots. The genotypes CQJ-5, Zhong R 2016, Liao 5856 and Xin lu zao 45 are located in front of the arrow in the figure, indicating that they had high yields. The genotypes Hongyemian and Ji zha 81 had lower yields, as indicated by their position behind the arrow. The stable genotypes included KK1543, Tianyun 10 and Zao 19, which showed relatively high yield and stability. Tu 76-94, Xi bu 50, Zhongmian 49 and Zhong R 2069 were identified as drought-sensitive genotypes. CQJ-5, Xin lu zao 45, Bellsno, Zhong R 2016 and ND 359-5 had high yields and stability (Fig. 4). This analysis was able to distinguish high-yielding and stable genotypes that can be used as high-yielding germplasm lines or for breeding ideal stable and high-yielding hybrids for cultivation under drought stress conditions. The results from the GGE biplots and the screening index analysis were basically the same, indicating the accuracy and effectiveness of the screening index.

## Discussion

### Identification of yield tolerance indices based on drought conditions

At present, water stress is the main abiotic stress factor that restricts crop growth (Boyer, 1982). In the cotton flowering and boll stages, water stress had a serious impact on cotton production (Kumar et al., 2008; Wang, Isoda & Wang, 2004). Therefore, cotton germplasm selection for varieties that require less water or tolerate water stress improves cotton adaptability to drought stress environments (Kumar et al., 2008). In the course of this study, seven screening indicators, MP, GMP, STI, SSI, Yr, Yp and Ys were used to identify the tolerance of 103 cotton germplasm lines to water stress (Table S6). The GMP indicates the average productivity of cotton material under water stress and normal conditions (Kristin et al., 1997). The MP indicates the average yield of cotton under water stress and normal conditions (Zhao et al., 2019). These two indicators reflect the average yield of cotton under different moisture conditions, but one of them provides a higher yield value and the other provides a lower yield value; this discrepancy may result in an incorrect assessment of the genotype (Table 1, Table S6). Therefore, using only the GMP and MP for production selection will lead to evaluation errors (Table S6). The results of Fernandez’s research under normal and stress conditions indicate that the STI can be used to evaluate high-yield germplasm lines (Fernandez, 1992). This indicator reflects the average yield of cotton material under stress and normal conditions (Table S6). Therefore, it was necessary to carry out a comprehensive appraisal and evaluation that included this index (Fig. 2). The genotype Xin lu zao 45 had lower MP and GMP values than the other tested varieties due to its lower yield, but it had a higher STI value, indicating that Xin lu zao 45 is a stable-yielding line (Table 1). There was a very significant positive correlation between the GMP, MP, and STI and cotton yield under normal and stress conditions, indicating that the GMP, MP and STI can collectively be used to distinguish varieties that produce high and stable cotton yields (Fig. 1). Other researchers have obtained similar research results in crops such as wheat and corn (Zhao et al., 2019). The results of other studies have indicated that there is a significant negative correlation between Yr and yield under water stress conditions (Fig. 1). Previous studies on low nitrogen tolerance have focused mainly on physiological or agronomic traits, including leaf area, sucrose content, biomass, etc (Duan et al., 2018). Different evaluation systems and processes have been proposed for use in different orders. The articles published on this topic thus far have proposed many screening indicators but have not reached a consensus on which indicators should be used.

### Analysis of the cotton drought tolerance evaluation system and selection of drought-tolerant germplasm

In this study, the combination of the cotton yield BLUP values and PCA was used to develop a drought tolerance index to evaluate cotton drought tolerance in terms of cotton yield, and five drought-tolerant cotton germplasm lines were screened (Table S6, Fig. 3). GGE biplots were used to analyze the genotypes for high yield and stable yield, and the results were consistent with the screening results based on yield screening indicators (Zhao et al., 2019). GGE biplots screen out adaptive germplasm lines under different conditions. At present, GGE biplots are rarely used in evaluations of cotton yield combined with drought resistance (Fig. 2). We bred the tested varieties under drought or water stress and selected germplasm lines with high and stable yields (Fig. 4). With the yield-based drought tolerance index, GGE biplots and 3D plots, the genotypes CQJ-5, Xin lu zao 45, Bellsno, Zhong R 2016 and ND 359-5 were identified as producing high yields under drought conditions (Fig. 3). These germplasm lines can provide a basis for our future development of high-yielding drought-tolerant cotton. Some researchers have identified the drought resistance of some cotton germplasm lines through multiple indicators, including plant height, number of fruit branches, and fiber quality. Among them, Xin lu zao 45 and Zhong R 2016 were identified as drought-resistant materials (Li et al., 2019). These two drought-resistant germplasm lines were also similarly identified by the yield traits in this study, indicating that these two germplasm lines do have good drought resistance.

## Conclusions

The results of this study show that the combination of the ST, MP and GMP is suitable for screening drought-tolerant genotypes because these indicators reflect the yield characteristics of each material under water stress. According to the yield 3D map and GGE biplots, CQJ-5, Xin lu zao 45, Bellsno, Zhong R 2016 and ND 359-5 were determined to be drought-tolerant genotypes. These genotypes could provide germplasm material for cultivating cotton varieties that produce high and stable yields under water stress conditions.

##  Supplemental Information

10.7717/peerj.14367/supp-1 Supplemental Information 1 Temperature and precipitation statistics from 2016 to 2018

10.7717/peerj.14367/supp-2 Supplemental Information 2 One hundred and three cotton varieties resources

10.7717/peerj.14367/supp-3 Supplemental Information 3 Soil water content in drought stress treatments (2016–2018)

10.7717/peerj.14367/supp-4 Supplemental Information 4 Analysis of variance for cotton plant yield BLUP (2016–2018)

10.7717/peerj.14367/supp-5 Supplemental Information 5 Results of the principal component analysis of the cotton yield per plant under normal (Yp) and stress (Ys) conditions and seven screening index values

10.7717/peerj.14367/supp-6 Supplemental Information 6 The linear unbiased prediction (BLUP) value of yield under normal (Yp) and stress treatments

10.7717/peerj.14367/supp-7 Supplemental Information 7 Raw data

## Additional Information and Declarations

### Data Availability

The following information was supplied regarding data availability: The raw measurements are available in the Supplementary Files.
